# Podophyllotoxin: a novel potential natural anticancer agent 

**Published:** 2017

**Authors:** Hamidreza Ardalani, Amir Avan, Majid Ghayour-Mobarhan

**Affiliations:** 1 *Department of Horticultural Sciences, Science and Research Branch, Islamic Azad University, Tehran, Iran*; 2 *Metabolic Syndrome Research Center, School of Medicine, Mashhad University of Medical Sciences, Mashhad, Iran*; 3 *Molecular Medicine Group, Department of Modern Sciences and Technologies, School of Medicine, Mashhad University of Medical Sciences, Mashhad, Iran*

**Keywords:** Podophyllotoxin, Anticancer, Antitumor, Natural products, Lignans

## Abstract

**Objective:**

The aim of the present review is to give an overview about the role, biosynthesis, and characteristics of Podophyllotoxin (PTOX) as a potential antitumor agent with particular emphasis on key biosynthesis processes, function of related enzymes and characterization of genes encoding the enzymes.

**Materials and Methods:**

Google scholar, PubMed and Scopus were searched for literatures which have studied identification, characterization, fermentation and therapeutic effects of PTOX and published in English language until end of 2016.

**Results:**

PTOX is an important plant-derived natural product, has derivatives such as etoposide and teniposide, which have been used as therapies for cancers and venereal wart. PTOX structure is closely related to the aryltetralin lactone lignans that have antineoplastic and antiviral activities. *Podophyllum emodi *Wall. (syn. *P. hexandrum*) and *Podophyllum peltatum* L. (Berberidaceae) are the major sources of PTOX. It has been shown that ferulic acid and methylenedioxy substituted cinnamic acid are the enzymes involved in PTOX synthesis. PTOX prevents cell growth via polymerization of tubulin, leading to cell cycle arrest and suppression of the formation of the mitotic-spindles microtubules.

**Conclusion:**

Several investigations have been performed in biosynthesis of PTOX such as cultivation of these plants, though they were unsuccessful. Thus, it is important to find alternative sources to satisfy the pharmaceutical demand for PTOX. Moreover, further preclinical studies are warranted to explore the molecular mechanisms of these agents in treatment of cancer and their possible potential to overcome chemoresistance of tumor cells.

## Introduction

**Figure 1:**
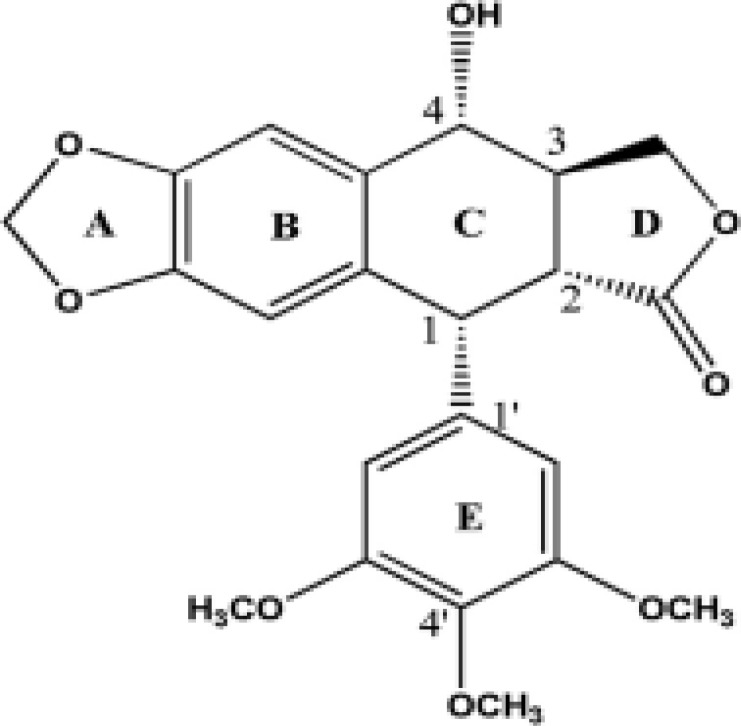
Chemical structure of podophyllotoxin

Extensive drug discovery and small molecules screening programs over the past thirty years, have presented plants and micro-organisms as rich sources of structurally diverse and highly bioactive natural products. One of the most important natural products are aryltetralin lactone lignans. Lignans are a family of natural products which are secondary metabolites produced through the shikimic acid pathway. The chemical structure of lignans is composed of two phenylpropane units and has a variety of skeletons and chemical characteristics; lignans can be divided into four groups: Lignans, Neolignans, Trimers and Oxyneolignans, higher analogues and mixed Lignanoids (Luo et al., 2014 ▶). It has been reported that natural aryltetralin lactone lignans are present in the plants of the families Cupressaceae, Berberidaceae, Apiaceae, Burseraceae, Verbenaceae, etc. Podophyllotoxin (PTOX) (C_22_H_22_O_8_) is an exclusive lignan because one of its derivatives was recognized as a potent antitumor factor (Figure 1) (Liu et al., 2015 ▶). Among the lignans, cyclolignans present a carbocycle between the two phenylpropane units, attached by two single carbon-carbon bonds through the side chains and one of them is between the β-βʹ positions. The aryltetralin structure of PTOX belongs to cyclolignans (Gordaliza et al., 2004 ▶). PTOX was first isolated in 1880 by Podwyssotzki from the North American plant *Podophyllum peltatum* L., commonly known as the American mandrake or May apple. This natural product has been also isolated from *Podophyllum emodi* (Indian podophyllum) (Ramos et al., 2001 ▶). It is particularly noticeable that PTOX is the most abundant lignan in podophyllin, a resin produced by species of the genus *Podophyllum* and some endophytic microorganisms in Cupressaceae family (Kusari et al., 2011 ▶). Despite extensive efforts done to propose new natural products for cancer therapy, a very small number of agents have been reached clinical practice. This can be explained at least in part by lack of enough knowledge about the molecular mechanisms of their action and possible side effects of these natural agents. Thus, the aim of current review is to give an overview about the role, biosynthesis, structure and spectral characteristics of aryltetralin lactone lignans as a potential antitumor agent.

**PTOX: origin and sources**

Lignans have been found in a large number of species, which are belonged to more than 60 families of vascular plants. Lignans can be isolated from different part of plants: roots and rhizomes, woody parts, stems, leaves, fruits, seeds and, in other cases, from endophytic microorganisms (Kusari et al., 2009 ▶; Schulz et al., 2002 ▶). Lignans have also been found in the urine of humans and mammals, although some of them are identical to components of the plant primary metabolites. Additionally, several distinct chemical reactions have been suggested, such as internal metabolic transformation (Zhang et al., 2014 ▶). It is important to mention that *Podophyllum *genus is not the only natural source of PTOX. This plant genus and other genera such as *Jeffersonia, Diphylleia* and *Dysosma *(Berberidaceae), *Catharanthus* (Apocynaceae), *Polygala* (Polygalaceae), *Anthriscus* (Apiaceae), *Linun* (Linaceae), *Hyptis* (Verbenaceae), *Teucrium*, *Nepeta* and *Thymus* (Labiaceae), *Thuja, Juniperus*, *Callitris* and *Thujopsis* (Cupressaceae), *Cassia* (Fabaceae), *Haplophyllum* (Rutaceae), *Commiphora* (Burseraceae), *Hernandia* (Hernandiaceae) can produce PTOX and derivatives (Table 1) (Kusari et al., 2011 ▶; Lim et al., 2007 ▶; Soltani and Moghaddam, 2015 ▶; Suzuki et al., 2002 ▶; Wink, 2012 ▶). However, environmental effects and difficulties in cultivation are the most marked problems in extraction of PTOX from plants (Dhar et al., 2002 ▶).

**Table 1:** The list of natural sources of PTOX reported in the literature (1996 – 2015).

**No.**	**Gander**	**Species**	**Family name**	**Part**	**References**
	Plant	*Anthriscus sylvestris* L.	Apiaceae	Root	(Jeong et al., 2007; Koulman et al., 2003)
		*Dysosma versipllis*	Berberidaceae	Root	(Yu et al., 1991)
		*Podophyllum hexandrum*	Berberidaceae	Root	(Chattopadhyay et al., 2002; Kumar et al., 2015)
		*Podophyllum versipelle* Hance.	Berberidaceae	Root	(Broomhead and Dewick, 1990b)
		*Sinopodophyllum emodi*	Berberidaceae	Leaf - Root	(Liang et al., 2015)
		*Juniperus sabina* L.	Cupressaceae	Leaf	(San Feliciano et al., 1989a)
		*Juniperus thurifera* L.	Cupressaceae	Leaf	(San Feliciano et al., 1989b)
		*Juniperus virginiana *L.	Cupressaceae	Leaf	(Kupchan et al., 1965)
		*Juniperus chinensis *L.	Cupressaceae	Root - Leaf	(Miyata et al., 1998; Muranaka et al., 1998)
		*Callitris intratropica*	Cupressaceae	Root - Leaf	(Wanner et al., 2015)
		*Juniperus horizontalis* Moench	Cupressaceae	Leaf	(Cantrell et al., 2014)
		*Juniperus scopulorum* Sarg.	Cupressaceae	Leaf	(Cantrell et al., 2013)
		*Juniperus virginiana* L.	Cupressaceae	Leaf	(Cushman et al., 2003)
		*Hyptis verticillata* Jacq.	Lamiaceae	Root - Leaf	(Kuhnt et al., 1994)
		*Nepeta nudaI *L.	Lamiaceae	Leaf - Root	(Konuklugil, 1996b)
		*Phlomis nissolii *L.	Lamiaceae	Leaf - Root	(Konuklugil, 1996b; Loike, 1982)
		*Salvia cilicica *Boiss.	Lamiaceae	Leaf - Root	(Konuklugil, 1996b)
		*Teucrium chamaedrys *L.	Lamiaceae	Leaf - Root	(Konuklugil, 1996b)
		*Teucrium polium* L.	Lamiaceae	Leaf - Root	(Konuklugil, 1996b)
		*Thymus capitatus* H.	Lamiaceae	Leaf - Root	(Konuklugil, 1996b)
		*Linum album *Kotschy ex Boiss.	Linaceae	Leaf - Root	(Chashmi et al., 2013; Yousefzadi et al., 2010)
		*Linum capitatum* Kit.	Linaceae	Root - leaf	(Broomhead and Dewick, 1990a)
		*Linum flavum* L.	Linaceae	Root - leaf	(Broomhead and Dewick, 1990a; Konuklugil, 1996a)
		*Linum thracicum*	Linaceae	Root - Leaf	(Sasheva and Ionkova, 2015)
		*Linum* *mucronatum* Gilib.	Linaceae	Root	(Samadi et al., 2014)
		*Polygala polygama *Walter.	Polygalaceae	Leaf	(Petersen and Alfermann, 2001)
	Fungi	*Mucor fragilis*	Mucoraceae	-	(Huang et al., 2014)
		*Mucor fragilis*	Mucoraceae	-	(Huang et al., 2014)
		*Fusarium oxysporum*	Nectriaceae	-	(Kour et al., 2008)
		*Fusarium oxysporum*	Nectriaceae	-	(Kour et al., 2008)
		* Fusarium solani*	Nectriaceae	-	(Nadeem et al., 2012)
		*Trametes hirsuta*	Polyporaceae	-	(Puri et al., 2006)
		*Trametes hirsuta*	Polyporaceae	-	(Puri et al., 2006)
		*Piriformospora indica*	Sebacinaceae	-	(Baldi et al., 2008)
		*Sebacina vermifera*	Sebacinaceae	-	(Baldi et al., 2008)
		*Piriformospora indica*	Sebacinaceae	-	(Baldi et al., 2008)
		*Aspergillus fumigatus*	Trichocomaceae	-	(Kusari et al., 2009)
			Vibrisseaceae	-	(Eyberger et al., 2006)

**PTOX synthesis**

**Figure 2:**
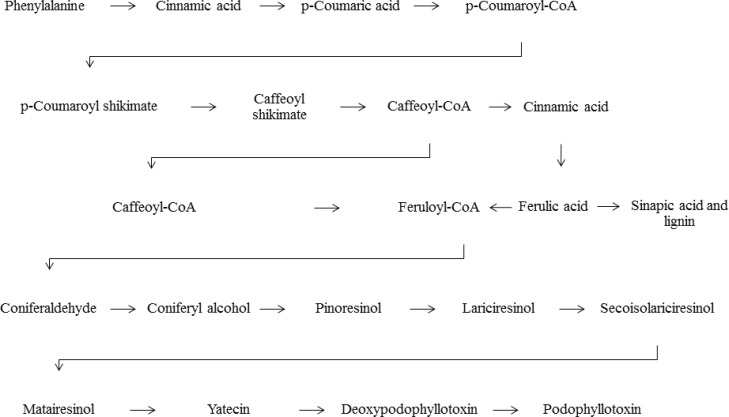
Synthesis pathway of podophyllotoxin

The complete biosynthetic pathway of PTOX has not been recognized so far, but several studies on some species of *Forsythia*, *Linum* and *Podophyllum *have documented some procedures for synthesis of PTOX (Petersen and Alfermann, 2001 ▶). It has been illustrated that ferulic acid and methylenedioxy substituted cinnamic acid are key enzymes involved in the biosynthesis of PTOX (Figure 2) and other natural cyclolignans (Khaled et al., 2013 ▶; Seidel et al., 2002 ▶). In an *in vitro* experiment on *Linum flavum *L., 6-methoxypodophyllotoxin was found as the main cyclolignan, although this compound was not detected when the cells were cultured with PTOX. The results of this study showed that high affinity of  kinetic constants reflects PTOX and PTOX can inhibit the production of 6-methoxypodophyllotoxin (Berim et al., 2008 ▶). Assessment of PTOX biosynthesis is a promising option which can help to discover novel sources and provide sustainable production of PTOX.

**Fermentation approach: a novel source of PTOX biosynthesis**

Several attempts have been made to develop and identify an alternative way for the production of aryltetralin lignans especially PTOX, although little success has been achieved. Among these technologies, fermentation technology is reported as a promising approach (Kesting et al., 2009 ▶). Nowadays, several natural products are produced by microorganisms such as paclitaxel from *Tubercularia* sp. (Wang et al., 2000 ▶), anti-cancer pro-drug PTOX from *Fusarium oxysporum *(Dar et al., 2013 ▶; Kour et al., 2008 ▶; Kumar et al., 2013 ▶). Increasing evidence has shown that host-microbe systems such as symbiosis living between fungi and/or plants can produce PTOX (Aly et al., 2010 ▶; Puri et al., 2006 ▶), including biotransformations with whole cell fermentations (Giri and Narasu, 2000 ▶; Puri et al., 2006 ▶). The approach used *Aspergillus fumigatus* (Trichocomaceae), an endophytic fungus from *Juniperus communis* L. Horstmann (Cupressaceae), that can biosynthesize aryl tetralin lignans, including PTOX (Kusari et al., 2009 ▶).

**Mechanism of action**

There is growing body of evidence showing the potential anti-cancer activity of PTOX. It has been shown that PTOX has anti-neoplastic properties that prevent the assembly of tubulin into microtubules and persuading apoptosis (Abad et al., 2012 ▶). This effect can be achieved by preventing the polymerization of tubulin which thereby could induce cell cycle arrest at mitosis and impede the formation of the mitotic-spindles microtubules. This mechanism of action is comparable with an another alkaloid, colchicine (Passarella et al., 2010 ▶). The antitumor activity of PTOX against lung metastatic cancer has been reported (Utsugi et al., 1996 ▶). In this study, the inhibitory activity of etoposide and PTOX against topoisomerase II via induction of DNA strand breaks was shown (Utsugi et al., 1996 ▶). The results of preclinical studies showed that PTOX prevented the polymerization of microtubule resulting in mitotic detention as shown by accumulation of mitosis-related proteins, BIRC5 and aurora B (Chen et al., 2013 ▶). PTOX reversibly binds to tubulin, and interrupts the dynamic equipoise between the assembly and disassembly of microtubules, and finally causes mitotic arrest (Guerram et al., 2012 ▶). Moreover, it has been shown that semi-synthetic products of PTOX, such as etoposide, teniposide and etopophos, have an inhibitory activity on DNA topoisomerase II that prevents the re-ligation of DNA (Choi et al., 2015 ▶; Shin et al., 2010 ▶; Xu et al., 2009 ▶) 

**Pharmacological activity of PTOX**

Several studies have shown the possible role of natural products in treatment of several disorders (Ardalani et al., 2016 ▶; Jandaghi et al., 2016 ▶) with different application to be used as antiviral, antifungal, antibacterial and anticancer agents. PTOX is suggested as an antiviral agent in the treatment of condyloma acuminatum caused by human papilloma virus (HPV) and other venereal warts (Wilson, 2002 ▶). In another *in vitro *study, podophyllotoxin derivatives showed promising cytotoxicities against a set of human cancer cell lines HL-60, A-549, HeLa, and HCT-8 (Liu et al., 2013 ▶). PTOX also activates pro-apoptotic endoplasmic reticulum stress signaling pathway. Intra-peritoneal injection of PTOX1 2 mg/kg significantly inhibited the growth of P-815, P-1537 and L-1210 tumor cells. The anti-tumor activity of this agent was more or less similar to that of paclitaxel (Wrasidlo et al., 2002 ▶). Also, the hematological and biochemical examinations showed that PTOX did not have organ toxicities in animal models (Chen et al., 2013 ▶). In a randomized clinical trial, the effects of PTOX on anogenital warts were compared with imiquimod 5% cream. The results showed a potent inhibitory effect on warts growth in PTOX-treated group vs. imiquimod cream-treated group (Komericki et al., 2011 ▶).

**Pharmacokinetic of PTOX**

In clinical studies, decreasing the absorption and the activity of a toxin in the living organism, is the major principles of poison management. Many clinical studies showed that dermal absorption of PTOX is limited and PTOX in body undergoes hepatic and renal metabolism (Lacey et al., 2003 ▶). In a study on anogenital warts, the topical application of 0.25 mg of PTOX was not associated with detectable serum concentrations of PTOX as measured by HPLC (Geo Von Krogh, 1982 ▶). Besides, it was found that the plasma concentration of PTOX, one hour after an intravenous injection of 3 mg/kg in rats, was indiscoverable and its excretion pathway is not clear yet (Deng et al., 1998 ▶).

**Clinical trials of PTOX**

The clinical trials on the use of PTOX and its derivatives against different sexual diseases, indicated that the use of this natural product could be effective on prevent and/or treatment of some cancer disorders. Most of clinical trials on PTOX were in treatment and control of genital warts and also the most effective time of treatment was 120 days (Table 2).

**Table 2:** Clinical studies on PTOX and its derivatives

**No.**	**Type of medication**	**Duration of study**	**No. of patients**	**Disease**	**References**
1	Cream and solution	90 days	358	genital warts	(Lacey et al., 2003)
2	Cream and solution	120 days	109	genital warts	(Beutner et al., 1989)
3	Cream	90 days	55	external anogenital warts	(Arican et al., 2004)
4	Cream	120 days	311	external anogenital warts	(Edwards et al., 1998)
5	Cream	60 days	108	genital warts	(Beutner et al., 1998)
6	Solution	30 days	38	penile warts	(Kirby et al., 1990)
7	Solution	160 days	57	penile warts	(von Krogh et al., 1994)
8	Cream	90 days	60	external genital condylomata acuminata	(Von Krogh and Hellberg, 1991)
9	Cream	180 days	150	molluscum contagiosum	(Syed et al., 1994)

## Conclusion

The use of the secondary metabolites of plants or microorganisms has gained substantial attention in the treatment of cancer. PTOX is being suggested as a potential anticancer agent in treatment of tumor cells via preventing the polymerization of tubulin which induces cell cycle detention at mitosis and prevents cancer cells development. PTOX derivatives, such as etoposide and teniposide, have been suggested to be used as therapies for cancers and venereal wart. One of the main problems with this plant-derived natural product is its resources, which has led to studies to identify new sources, such as cultivation, plant cell or organ culture, and chemical synthesis. Therefore, understanding biosynthesis of PTOX is a key step for cultivation of medicinal plants. Indeed, we can use of microorganisms as an alternative source of PTOX. According to previous studies, synthesis of lignans in different microorganisms is not the same. Also, research on the synthesis pathways of PTOX in different microorganisms might be helpful to find the best source of this valuable lignin.
